# Personality traits and Chinese college students’ satisfaction with physical education classes: the mediating role of trait fluency and the moderating role of physical education class difficulty

**DOI:** 10.3389/fpsyg.2023.1270089

**Published:** 2023-12-20

**Authors:** Zhiduo Chen, Yuge Tian, Min Li, Shangjian Yang

**Affiliations:** ^1^School of Physical Education, Shandong University, Jinan, China; ^2^School of Physical Education, Shanghai Normal University, Shanghai, China

**Keywords:** personality trait, trait fluency, student satisfaction, personality factors, difficulty and flow experience, moderated mediation

## Abstract

**Background:**

This study aims to investigate the impact mechanism of personality traits on physical education satisfaction among college students, validating the mediating effect of trait flow and the moderating effect of physical education difficulty. By analyzing the influence mechanism of personality traits on college students’ satisfaction with physical education classes, it helps to explore more channels to enhance satisfaction with physical education classes.

**Methods:**

A questionnaire survey was conducted using the Big Five Personality Scale, the Physical Education Class Satisfaction Scale, the Trait Fluency Scale, and the Physical Education Class Difficulty Scale with 868 public physical education students in 10 universities in Shanghai. Moderated mediation modeling was conducted using Hayes’ PROCESS macro.

**Results:**

Personality traits are positively correlated with physical education satisfaction, and the predictive effect is significant (*β* = 0.786, *p* < 0.001). This association is mediated by trait fluency (indirect effect: *β* = 0.797, *p* < 0.001), accounting for 62.7% of the total effect. Physical education difficulty significantly moderates the predictive effects of personality traits on physical education satisfaction (*β* = −0.183, *p* < 0.01) and trait fluency (*β* = −0.130, *p* < 0.001). Additionally, physical education difficulty significantly moderates the predictive effect of trait fluency on physical education satisfaction (*β* = 0.172, *p* < 0.001).

**Conclusion:**

Personality traits predict physical education satisfaction, with trait fluency playing a mediating role, and physical education difficulty moderates the direct and indirect paths through which personality traits influence physical education satisfaction.

## 1 Introduction

In China, school physical education, as one of the key tasks of educational modernization (General Office of the CPC Central Committee, 2019), has received significant attention. Over the past decade, various Chinese central government departments have jointly issued as many as 18 working documents related to school physical education (Liu et al., 2023). These documents aim to provide guidance for school physical education and promote physical education programs (Chen and Wang, 2022). The primary objective of school physical education has always been to enhance students’ physical fitness (Zhang, 2015). Physical education courses are considered a crucial component of the Chinese education system (Ma, 2020), spanning from primary and secondary schools to universities (Wang, 2012). Due to the impact of COVID-19, physical education programs have been variably affected, leading to a reduction in physical activities among college students (Cha, 2022). In recent years, the physical well-being of Chinese university students has witnessed a persistent decline (Chen et al., 2020; Education, 2021; Yu et al., 2022). As of the year 2020, the proportion of Chinese university students failing to meet the standard of physical fitness stands at 30% (Wu and Ji, 2023). Moreover, there exists a marked indifference toward sports consciousness and interests, coupled with a lack of awareness and capacity for self-health management (Baena-Extremera et al., 2012). Public physical education classes represent the primary bastion for college students’ physical exercise and the cultivation of sporting interests (He, 2019), high quality physical education classes can improve students’ physical fitness and health (Kavoura et al., 2021). Measuring satisfaction has become a “hot topic” in higher education research. Previous research has demonstrated the importance of satisfaction in physical education classes, which is critical to improving students’ physical health, motor skills, mental health, and social competence (White et al., 2021; Correa-Barwick et al., 2022). This is reinforced by the above mentioned physical fitness status of college students, thus there is a great need to explore the current status of college students’ satisfaction with physical education classes as well as the direct and indirect factors.

Research has indicated that personality traits have an impact on students’ academic satisfaction (Bolliger and Erichsen, 2013; Kim et al., 2013; Shih et al., 2014). Sutin’s study found a significant correlation between personality traits and engagement in physical activities, with personality traits either facilitating or hindering such involvement (Sutin et al., 2016). Evaluating personality traits allows for a relatively accurate prediction of students’ levels of satisfaction with their academic experiences (Baruth and Cohen, 2023). Personality traits cover a wide range, and having the extroverted quality of cheerfulness and friendliness produces positive emotions after physical activity (Huang et al., 2007; Wichers et al., 2012). Additionally, personality traits that exhibit emotional richness, creativity, conscientiousness, and dutifulness have been confirmed to positively correlate with course satisfaction (Cohen and Baruth, 2017; Kavoura et al., 2021) and significantly predict satisfaction with courses (Baruth and Cohen, 2023). Furthermore, research has shown that traits such as agreeableness and humility positively influence course satisfaction (Baruth and Cohen, 2023), but some studies have yielded conflicting conclusions, finding no significant relationship (Downs, 2019; Kavoura et al., 2021). Taking Baruth’s study as an example, it explores the relationship between personality traits and course satisfaction among 108 current university students at Israel (Baruth and Cohen, 2023). However, prior research has not examined the mediating and moderating mechanisms, leaving room for further investigation in this study. Thus far, there has been no direct research investigating the relationship between personality traits and satisfaction with physical education classes, and the underlying potential mediation and moderation mechanisms remain largely unknown. The present study seeks to explore the connection between personality traits and satisfaction with physical education classes, while examining the potential mediating and moderating roles at play.

On the other hand, despite the prevalent use of linear models to explore the relationship between personality and satisfaction, it is imperative to investigate the inconsistencies by employing mediator and moderator variables (Hou et al., 2018). The presence of mediator or moderator variables adds complexity to the relationship, but theoretically provides a more accurate explanation of the observed phenomena. Among these variables, flow experience describes a state in which individuals wholeheartedly immerse themselves in an activity, experiencing a high level of satisfaction and enjoyment (Beck, 1992). Jackson distinguish flow experience into state fluency and trait fluency. State fluency pertains to the psychological state in which individuals experience flow in specific circumstances, whereas trait fluency represents a personality trait, reflecting an individual’s capacity to experience flow. Individuals with higher levels of trait fluency are more prone to experiencing flow states (Jackson et al., 1998). Engaging in physical activities can evoke the experience of trait fluency (Kawabata, 2018; Yoon et al., 2020), with each physical activity having the potential to induce trait flow in its unique way (Elkington, 2011). On the one hand, college students’ trait fluency in physical education classes influences their satisfaction with these classes (Hu et al., 2008). On the other hand, recent studies have demonstrated that trait fluency plays a mediating role in physical activities (Duan et al., 2022; Lin, 2023; Qiu et al., 2023), with individuals high in trait flow more inclined to persevere in exercise and establish a regular exercise routine (Qu et al., 2017). Therefore, trait fluency may act as a mediating variable connecting personality traits to satisfaction with physical education classes.

Furthermore, research has provided evidence of a significant correlation between course difficulty and satisfaction (Lee and Nuatomue, 2021). In a study investigating factors influencing student satisfaction in British business schools, Sutherland found that increasing levels of academic difficulty negatively impact satisfaction, leading to a decline in overall satisfaction (Sutherland et al., 2019). Another study showed that occupational difficulty significantly affects life satisfaction, and college students’ employment difficulty can negatively affect satisfaction (Cheng et al., 2014). Thus, it is evident that course difficulty plays a pivotal role in course satisfaction (Tu, 2007). However, there has been a dearth of research examining course difficulty as a moderating variable to explore its relationship with personality traits and course satisfaction. Consequently, this study aims to investigate the moderating role of course difficulty and analyze its influence on the relationship between personality traits and satisfaction with physical education classes.

**Figure 1:**
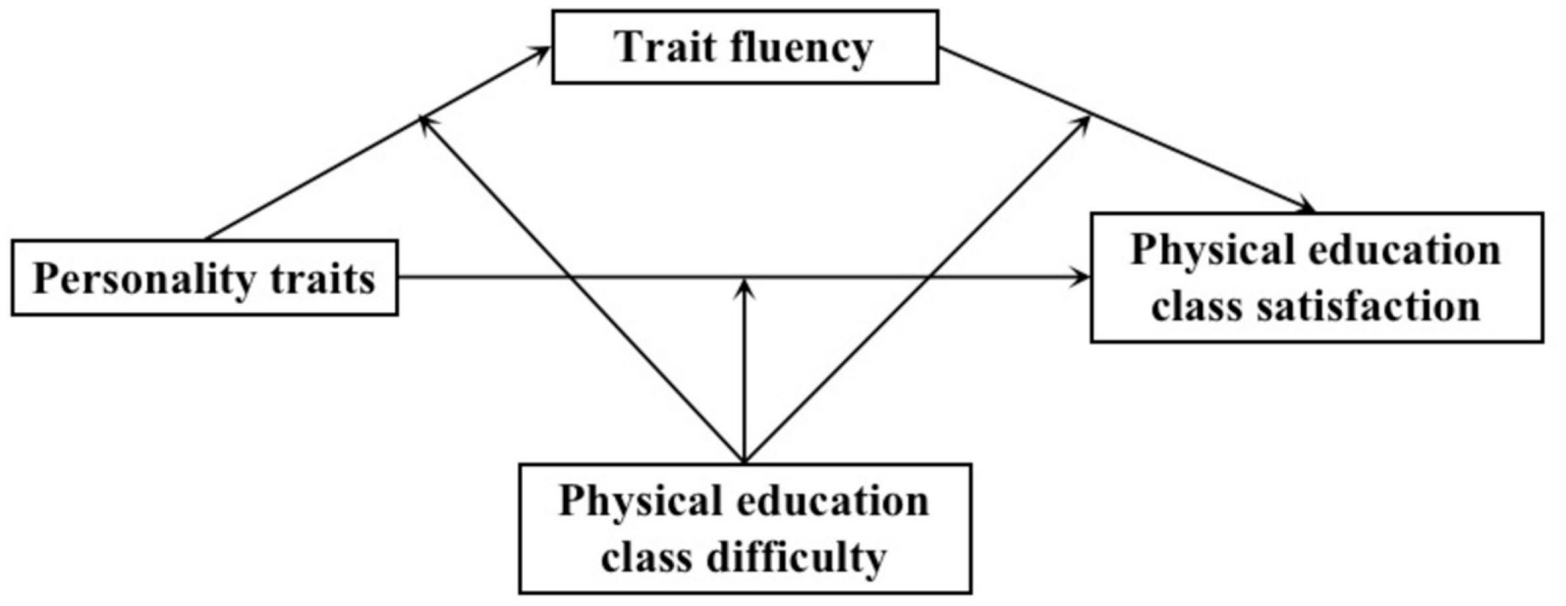
Theoretical model.

Drawing upon the aforementioned literature, we have proposed a theoretical model, as depicted in Figure 1, with the aim of exploring the interplay between personality traits, satisfaction with physical education classes, trait fluency, and physical education difficulty. Our objective is to analyze the mechanisms through which personality traits influence satisfaction with physical education classes and to validate the mediating effect of trait fluency and the moderating effect of physical education difficulty. Specifically, we have put forth the following hypotheses:

*Hypothesis 1*: There is a positive correlation between personality traits and satisfaction with physical education classes.

*Hypothesis 2*: Trait fluency serves as a mediating mechanism in the relationship between personality traits and satisfaction with physical education classes.

*Hypothesis 3*: The direct and indirect relationships between personality traits and satisfaction with physical education classes are both influenced by the moderating effect of physical education difficulty.

## 2 Methods

### 2.1 Participants

In the preliminary pre-survey, 80 questionnaires were distributed to students at Shanghai Normal University. Eighteen questionnaires were excluded due to missing key information and items, resulting in 62 valid questionnaires. In the subsequent main survey, the qualified questionnaires from the pilot survey were used, and a convenient sampling method was employed to survey college students from 10 universities in Shanghai. Both online electronic questionnaires and offline paper-based questionnaires were distributed. Only participants who provided informed consent were allowed to proceed with the survey. The participants in this study were students from various universities and majors, representing diverse academic backgrounds, including computer science, literature, and arts colleges, among others. Inclusion criteria were as follows: (1) undergraduate students, (2) students who had completed a certain number of physical education courses, and (3) students in good physical health. Exclusion criteria were: (1) non-undergraduate students, (2) students with severe health issues or injuries, and (3) students who had not participated in a sufficient number of physical education courses. A total of 957 questionnaires were distributed, with 489 being online surveys and 468 in paper format. Sixty-nine online surveys and twenty paper surveys were excluded due to incomplete data. This resulted in 868 valid questionnaires, with an effective response rate of 90.7%. According to research standards, a sample size between 5 to 10 times the number of items on the questionnaire is acceptable (Fritz and MacKinnon, 2007). In this study, there were a total of 43 items, so a sample size exceeding 215 is adequate. The final sample size for this study was 868, meeting the requirements. The average age of the participants was 19.29 ± 1.26 years, with 33% being male and 67% female. The participation rates of participants from each university were approximately 10%.

### 2.2 Measurement

#### 2.2.1 Big five personality scale

This study employed the Brief Form of the Chinese Adjective-Based Big Five Personality Inventory (BFFP-CAS-S), devised by Luo and Dai (2018), to assess the Big Five personality traits. The inventory consists of 20 items, encompassing five individual dimensions: extraversion, agreeableness, conscientiousness, neuroticism, and openness. Examples of questions are “I am often afraid,” “I feel like I am going to break down when I am under pressure” and “I have quite an imagination.” Each item in the scale was rated on a 5-point Likert scale, ranging from “strongly disagree” (1) to “strongly agree” (5). Previous research has substantiated the BFFP-CAS-S to demonstrate sound reliability and validity (Luo et al., 2019). The Cronbach’s alpha coefficient for the entire scale was calculated to be 0.86.

#### 2.2.2 Physical education class satisfaction scale

This study employed the Adolescent Physical Exercise Satisfaction Scale, revised by Yang (2016) which is an adaptation of the Satisfaction with Life Scale originally developed by Diener et al. (1985). In this study, the term “physical exercise” was replaced by “physical education class” to form a unidimensional structure consisting of five entries, including: (1) “Most aspects of my physical education class are close to my ideal.” (2) “My state during physical education class is very good.” (3) “I am satisfied with my physical education class.” (4) “So far, I have obtained important things I wanted from physical education class.”(5) “If I could go back and choose my physical education class again, I would hardly change anything.” Each item in the scale was rated on a 5-point Likert scale, ranging from “strongly disagree” (1) to “strongly agree” (5), with higher scores indicating higher satisfaction with physical education classes. The Cronbach’s alpha coefficient for the entire scale was calculated to be 0.90 (See Supplementary material).

#### 2.2.3 Trait fluency scale

This study used Liu’s (2010) simplified single-dimension multi-item Trait Fluency Scale to evaluate participants’ general inclination to experience flow characteristics. Participants were asked to recall the frequency with which they typically experience each flow-related item during physical education classes. The scale consists of 9 items, corresponding to 9 flow characteristics: 1 = balance between challenge and skill; 2 = integration of action and awareness; 3 = clear goals; 4 = immediate feedback; 5 = concentration; 6 = sense of control; 7 = loss of self-consciousness; 8 = altered sense of time; 9 = autotelic experience. Each item on the scale was rated using a 5-point Likert scale, ranging from “never” (1) to “always” (5). The overall scale demonstrated excellent internal consistency, with a Cronbach’s alpha coefficient of 0.90.

#### 2.2.4 Physical education difficulty scale

In this study, the MOOC Difficulty Scale, revised by Zhang (2019), was employed, with the term “MOOC” replaced by “physical education classes,” resulting in a unidimensional structure consisting of three items to assess the difficulty level of physical education classes. This scale was used to measure students’ evaluation of the difficulty level in physical education classes, including: “I find completing gym class to be difficult,” “Completing physical education classes is a challenge for me.” and “I find PE very complicated.” Each item on the scale was rated using a 5-point Likert scale, ranging from “strongly disagree” (1) to “strongly agree” (5), with higher scores indicating higher difficulty in physical education classes. The Cronbach’s alpha coefficient for the entire scale was calculated to be 0.87 (See Supplementary material).

### 2.3 Procedures

**Figure 2:**
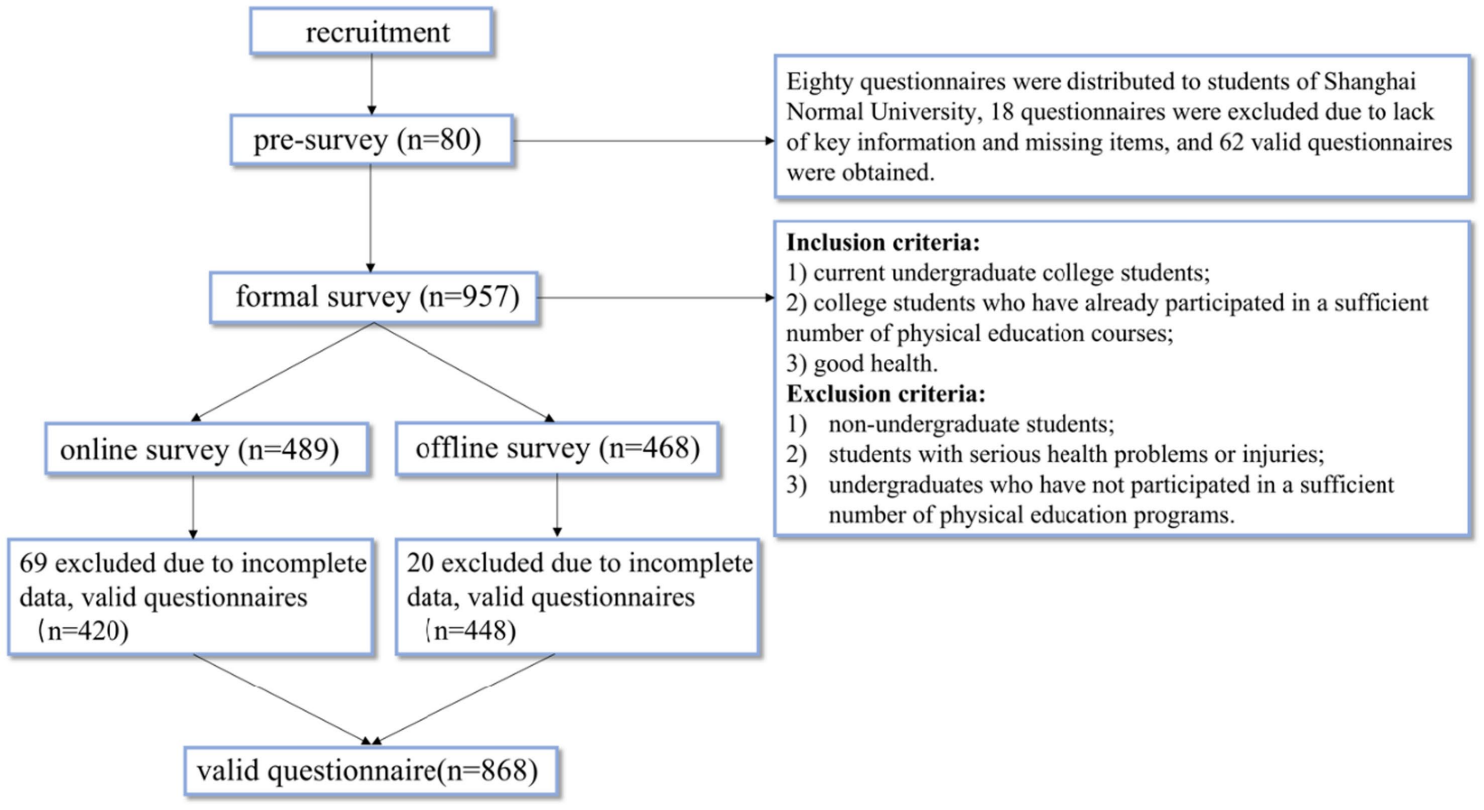
Data collection flowchart.

This study illustrates the specific steps and procedures of data collection (Figure 2). Data for the pilot survey and the formal survey were collected from February to March 2020 within university settings using a combination of online and offline methods. Offline data collection was carried out by researchers through on-site surveys at various universities in Shanghai, where they interacted with participants and addressed their inquiries. Online data were collected using the secure online survey platform “Wenjuanxing” (https://www.wjx.cn). Our survey was conducted in an environment of independent completion, without the presence of teachers waiting nearby. We provided the questionnaire link to students and promoted it in the classroom, encouraging them to complete the survey within the specified time. This design was implemented to maximize the autonomy and independence of the participants. Trained researchers administered these measures using scripts and procedural manuals to ensure standardization in the data collection process. Prior to data collection, informed consent forms were distributed to university students, and all students provided their consent for the handling of their relevant data in association with their participation in the study. Students were informed that their participation was entirely voluntary, and they could opt out at any time.

### 2.4 Statistical analysis

All statistical analyses were conducted using SPSS 23.0 software. Initially, descriptive statistics (i.e., M, SD) were computed for all variables, followed by bivariate correlations among these variables, with *p* < 0.05 (two-tailed) set as the significance level. Moderated mediation analysis was performed using the SPSS-Process 4.1 developed by Hayes. The association between personality traits and satisfaction with physical education classes was mediated by trait flow. Model 4 was employed to examine the mediated relationship between personality traits and satisfaction with physical education classes through trait flow. The moderating effect of physical education class difficulty on the mediation model was tested using Model 59, employing 5,000 bootstrap samples and bias-corrected 95% confidence intervals for bootstrap analysis. All models controlled for covariates such as age, gender, and school.

## 3 Results

### 3.1 Descriptive statistics

The results of the descriptive statistical analysis (Table 1) reveal that the mean score for satisfaction with physical education classes is 3.50 ± 0.89, indicating that college students’ satisfaction with physical education classes is at a moderately high level.

**Table 1:** Descriptive statistics of college students’ satisfaction with physical education classes (overall score situation).

	*N*	Minimum value	Maximum values	*M*	*SD*
PE satisfaction	868	1	5	3.507	0.893

PE, physical education.

The demographic characteristics are presented in detail in Table 2. There were no significant differences in university students’ satisfaction with physical education courses regarding gender (*p* > 0.05). However, significant differences were observed in terms of grade level (*t* = 5.923, *p* < 0.05) and school (*t* = 24.751, *p* < 0.01). Concerning grade level, sophomore students had a higher mean satisfaction score (*M* = 3.583), while senior students had a lower mean score (M = 3.195). Regarding schools, satisfaction with physical education courses was higher at Shanghai Normal University (*M* = 3.923) and lower at Shanghai Sports University (*M* = 3.325).

**Table 2:** Analysis of differences in satisfaction across demographic variables (*N* = 868).

Variant		Number	*M*	*SD*	*t/F*	*p*
Gender	Male	283	3.444	0.928	−1.453	0.146
	Female	585	3.538	0.875		
Grade	Freshman	331	3.561	0.881	5.923	0.001
	Sophomore	345	3.583	0.899		
	Junior	113	3.336	0.891		
	Senior	79	3.195	0.838		
School	1. Fudan University	81	3.632	0.846	24.751	0.000
	2. Shanghai Jiao Tong University	85	3.612	0.806		
	3. East China Normal University	95	3.579	0.755		
	4. Donghua University	79	3.635	0.688		
	5. Shanghai University	97	3.658	0.902		
	6. Shanghai Normal University	80	3.923	0.893		
	7. Shanghai University of Traditional Chinese Medicine	97	3.773	0.785		
	8. Shanghai University of Engineering and Technology	87	3.554	0.902		
	9. Shanghai University of Applied Sciences	87	2.368	0.477		
	10. Shanghai Sports Institute	80	3.325	0.852		

### 3.2 Correlation matrix of physical education class satisfaction with other variables

The results of correlation analysis (see Table 3) indicate that personality traits are significantly positively correlated with satisfaction with physical education classes and trait fluency. Moreover, trait fluency is significantly positively correlated with satisfaction with physical education classes. On the other hand, there is a significant negative correlation between the difficulty of physical education classes and both satisfaction with physical education classes and trait fluency. Thus, Hypothesis 1 is supported.

**Table 3:** Analysis of relevant results (*N* = 868).

Variant	*M*	*SD*	1	2	3	4
1. Personality traits	3.312	0.561	1			
2. PE satisfaction	3.507	0.893	0.517^**^	1		
3. Trait fluency	3.318	0.746	0.620^**^	0.642^**^	1	
4. PE difficulty	2.478	0.989	0.126^**^	0.167^**^	0.075^**^	1

^**^At the 0.01 level (two-tailed), the correlation was significant.

### 3.3 Testing for mediation effect

After controlling for gender, age, and school as covariates, the mediating effect of trait fluency in the relationship between personality traits and satisfaction with physical education classes was examined. The results are presented in Table 4. It was found that personality traits significantly predicted satisfaction with physical education classes (*β* = 0.786, t = 16.409, *p* < 0.001). Furthermore, even after introducing trait fluency as a mediating variable, the direct predictive effect of personality traits on satisfaction with physical education classes remained significant (*β* = 0.293, *t* = 5.564, *p* < 0.001). Additionally, the predictive effect of personality traits on satisfaction with physical education classes remained significant under the mediation of trait fluency (*β* = 0.797, *t* = 21.845, *p* < 0.001), and trait fluency also significantly predicted satisfaction with physical education classes (*β* = 0.618, *t* = 15.677, *p* < 0.001).

**Table 4:** Mediation model test for trait fluency (*N* = 868).

Regression equation		Goodness of fit index			Significance of the coefficient		
Outcome variable	Predictor variable	*R*	*R* ^2^	*F*	*β*	*t*	*p*
Satisfaction	Gender	0.527	0.277	82.870	−0.039	−0.707	0.480
	Grade				−0.042	−1.480	0.139
	School				−0.027	−2.809	0.005
	Personality trait				0.786	16.409	0.000
Trait fluency	Gender	0.630	0.397	142.043	−0.077	−1.813	0.070
	Grade				−0.030	−1.386	0.166
	School				−0.024	−3.244	0.001
	Personality trait				0.797	21.845	0.000
Satisfaction	Gender	0.662	0.438	134.210	0.008	0.166	0.868
	Grade				−0.024	−0.936	0.349
	School				−0.012	−1.443	0.150
	Personality trait				0.293	5.564	0.000
	Trait fluency				0.618	15.677	0.000

Subsequently, based on bootstrap confidence intervals, the direct effects of personality traits on physical education class satisfaction, as well as the upper and lower bounds of the mediating effect of trait fluency, were found to be non-inclusive of 0 (Table 5). The direct effect (0.293) accounted for 37.301% of the total effect (0.768), while the mediating effect (0.493) accounted for 62.712% of the total effect (0.768). These findings indicate that personality traits not only have a direct predictive effect on physical education class satisfaction but also exert their predictive influence on it through the mediating role of trait fluency. Therefore, Hypothesis 2 is supported.

**Table 5:** Breakdown of total, direct, and mediating effects.

	Effect value	Boot standard error	Boot ULCI	Boot LLCI	Relative effect value
Total effect	0.786	0.048	0.692	0.880	/
Direct effect	0.293	0.053	0.190	0.396	37.301%
Mediating effects	0.493	0.044	0.411	0.581	62.712%

ULCI, upper level of confidence interval; LLCI, lower level of confidence.

### 3.4 Tests for moderated mediation effects

After controlling for covariates such as gender, age, and school, we examined the moderating effect of physical education class difficulty on the direct and indirect relationship between personality traits and physical education class satisfaction. The results revealed (Tables 6, 7) significant predictive effects of the interaction term between personality traits and physical education class difficulty on physical education class satisfaction (*β* = −0.183, *t* = −3.849, *p* < 0.01), as well as on trait fluency (*β* = −0.130, *t* = −3.801, *p* < 0.001). Additionally, the interaction term between trait fluency and physical education class difficulty significantly predicted physical education class satisfaction (*β* = 0.172, *t* = 5.201, *p* < 0.001). These findings suggest that physical education class difficulty not only moderates the direct prediction of personality traits on physical education class satisfaction but also moderates the prediction of personality traits on trait fluency and trait fluency on physical education class satisfaction. Therefore, Hypothesis 3 is supported.

**Table 6:** Mediation model test with moderation (*N* = 868).

Regression equation		Goodness of fit index			Significance of the coefficient		
Outcome variable	Predictor variable	*R*	*R* ^2^	*F*	*β*	*t*	*p*
TF	Gender	0.644	0.414	101.556	−0.083	−1.965	0.050
	Grade				−0.022	−1.001	0.317
	School				−0.025	−3.426	0.001
	PT				0.786	21.635	0.000
	PE difficulty				−0.048	−2.386	0.017
	PT × PE difficulty				−0.130	−3.801	0.000
Satisfaction	Gender	0.692	0.479	98.582	0.017	0.361	0.718
	Grade				−0.018	−0.738	0.461
	School				−0.017	−2.029	0.043
	PT				0.361	6.999	0.000
	Trait fluency				0.574	14.857	0.000
	PE difficulty				−0.144	−6.218	0.000
	PT × PE difficulty				−0.183	−3.849	0.000
	TF × PE difficulty				0.172	5.201	0.000

PT, personality trait; TF, trait fluency.

**Table 7:** Moderated mediating effects of difficulty in physical education classes across levels of moderation.

	PE difficulty	Effect value	Boot standard error	Boot ULCI	Boot LLCI
MME	1.48 (M-1SD)	0.370	0.070	0.505	0.232
	2.47 (M)	0.451	0.043	0.537	0.368
	3.46 (M + 1SD)	0.490	0.070	0.623	0.345

MME, moderated mediation effect; Boot standard error refers to the standard error of the indirect effect estimated by the bias-corrected percentile Bootstrap method, Boot CI upper and lower bounds refer to the upper and lower bounds of the 95% confidence interval; all values are retained to two decimal places based on rounding.

**Figure 3:**
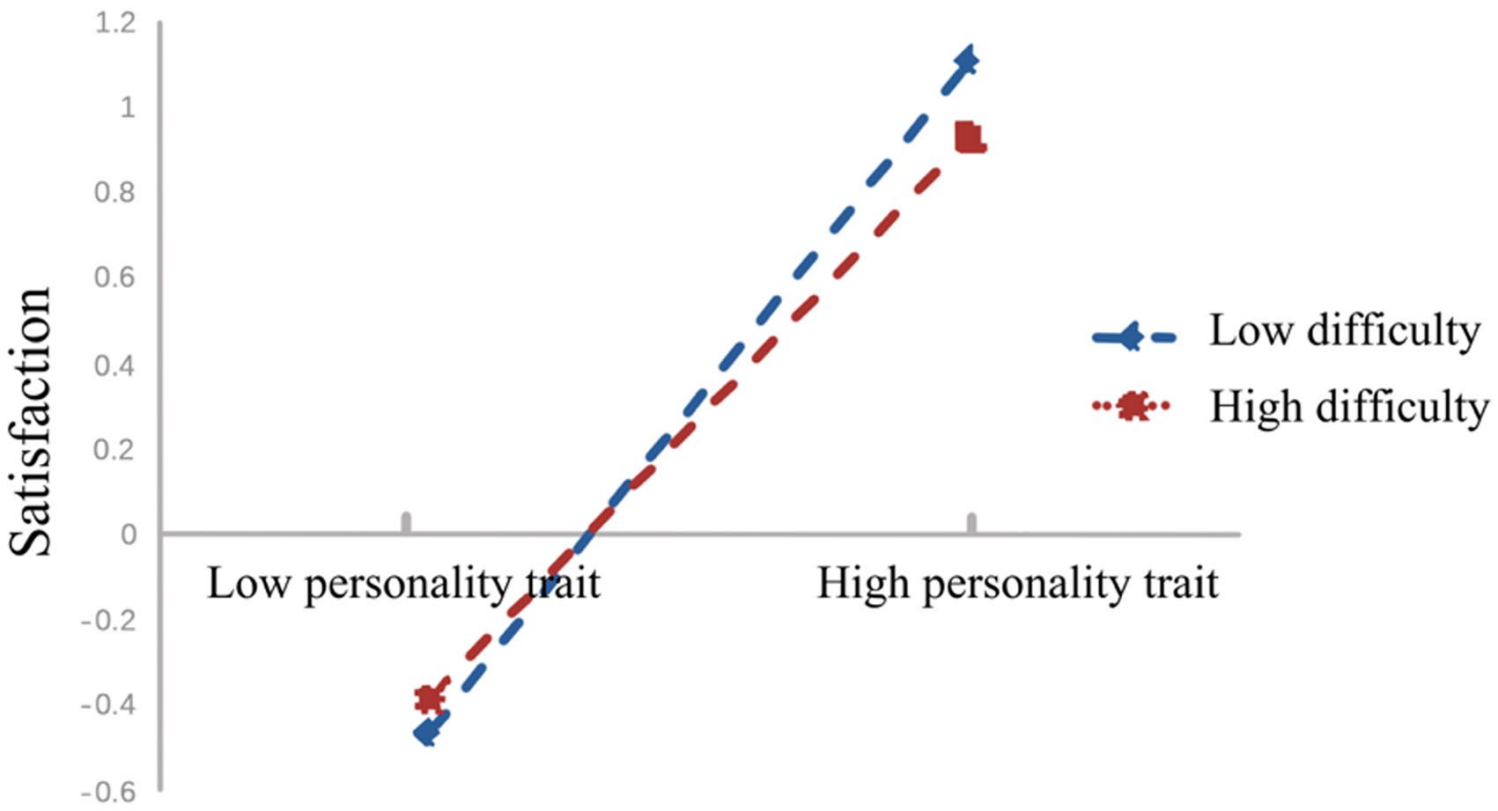
Moderating role of physical education class difficulty in the relationship between personality traits and trait fluency.

**Figure 4:**
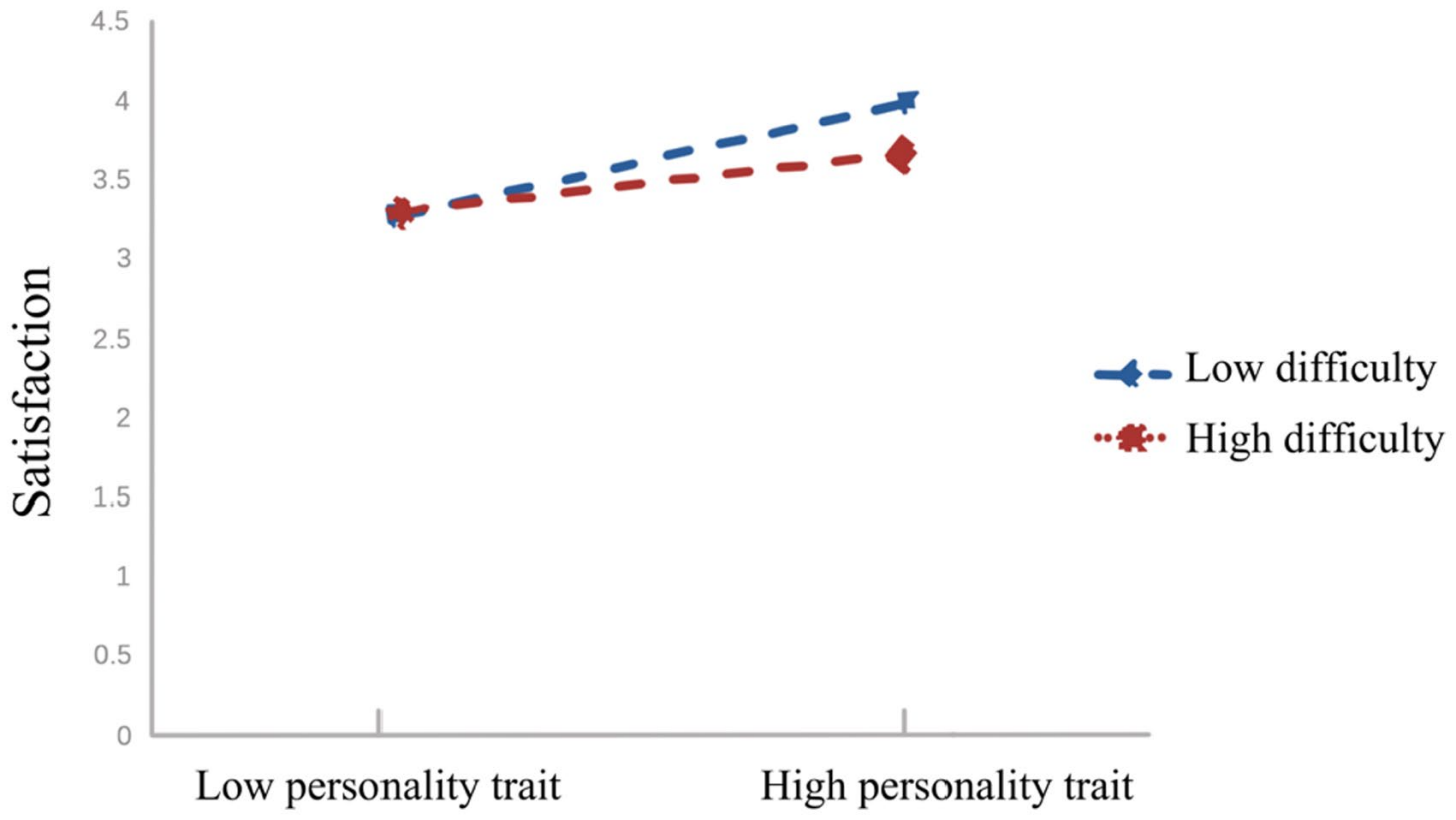
Moderating role of physical education class difficulty in the relationship between personality traits and physical education class satisfaction.

To reveal specific moderating effects, participants were divided into “low group” and “high group” based on M ± 1SD, and further subjected to simple slope analysis (see Figures 2–4). From Figure 2, it can be observed that for participants with lower levels of physical education class difficulty (M−1SD), personality traits significantly positively predicted trait fluency (*βsimple* = 0.91, *p* < 0.001). Similarly, for participants with higher levels of physical education class difficulty (M + 1SD), personality traits also positively predicted trait fluency (*βsimple* = 0.66, *p* < 0.001), but the magnitude of this prediction was relatively smaller. This suggests that with the increase in physical education class difficulty, the predictive effect of personality traits on trait fluency gradually diminishes.

From Figure 3, it can be observed that for participants with lower levels of physical education class difficulty (M-1SD), personality traits significantly positively predicted physical education class satisfaction (*βsimple* = 0.54, *p* < 0.001). Similarly, for participants with higher levels of physical education class difficulty (M + 1SD), personality traits also positively predicted physical education class satisfaction (*βsimple* = 0.18, *p* < 0.001), but the magnitude of this prediction was relatively smaller. This suggests that with the increase in physical education class difficulty, the predictive effect of personality traits on physical education class satisfaction gradually diminishes.

**Figure 5:**
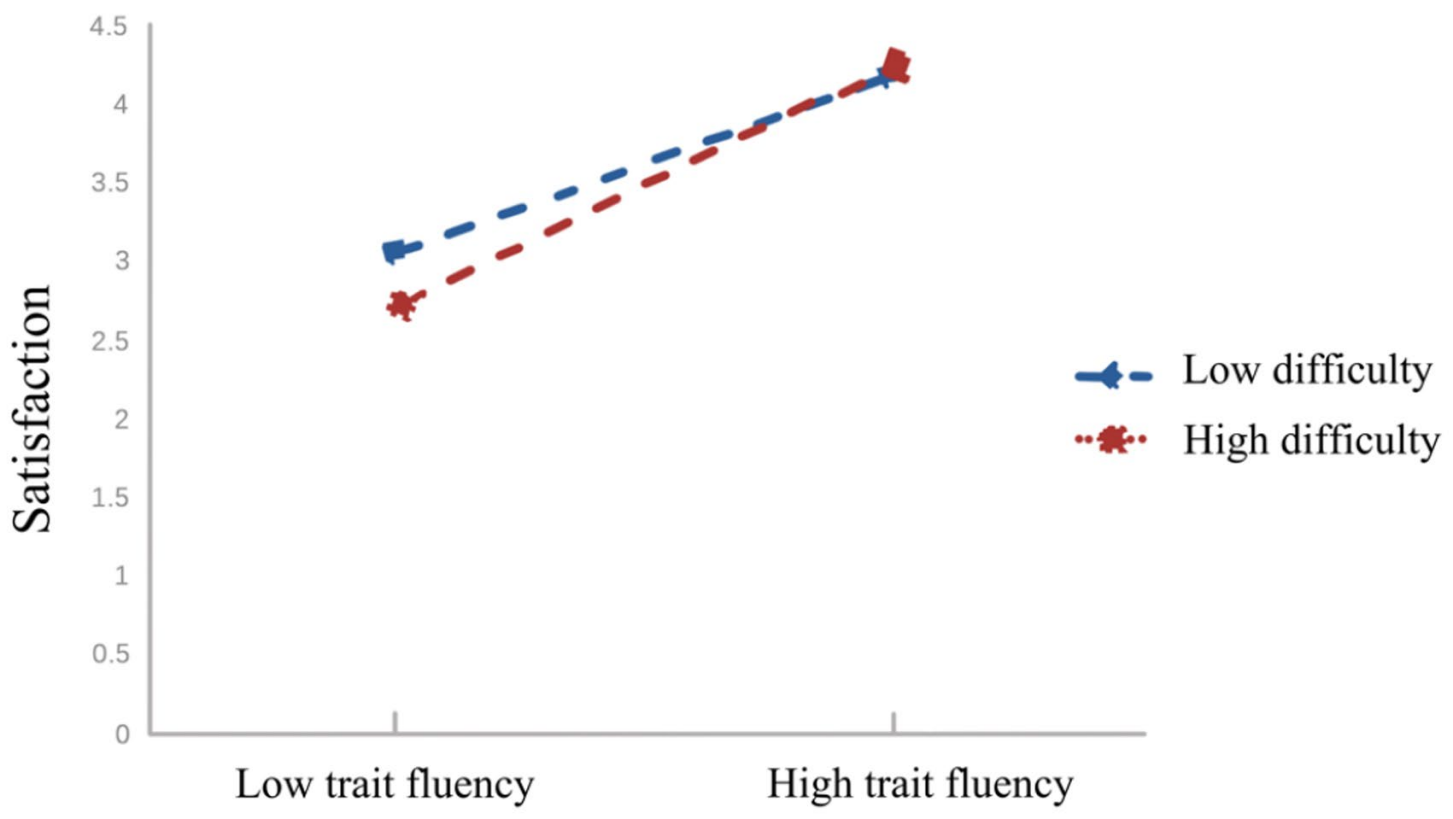
Moderating role of physical education class difficulty in the relationship between trait fluency and physical education class satisfaction.

Based on Figure 5, it is evident that for participants with lower levels of physical education class difficulty (M-1SD), trait fluency positively predicted physical education class satisfaction (*βsimple* = 0.40, *p* < 0.001), but the predictive effect was relatively smaller. Conversely, for participants with higher levels of physical education class difficulty (M + 1SD), trait fluency also positively predicted physical education class satisfaction, and the predictive effect was relatively larger (*βsimple* = 0.75, *p* < 0.001). This suggests that with the increase in physical education class difficulty, the predictive effect of trait fluency on physical education class satisfaction shows a gradual upward trend.

## 4 Discussion

This study conducted an investigation on college students’ participation in public physical education classes, aiming to explore the mediating role of trait fluency and the moderating effect of physical education class difficulty in the relationship between personality traits and physical education class satisfaction. The research findings have provided valuable data and contributed to a deeper understanding of the factors related to physical education class satisfaction with respect to personality traits.

The importance of personality traits as influential factors on course satisfaction has been empirically supported (Bolliger and Erichsen, 2013; Shih et al., 2014). In this study, we further refined course satisfaction to specifically focus on physical education class satisfaction, and the results revealed a positive correlation between personality traits and physical education class satisfaction. Moreover, personality traits were found to have a predictive effect on physical education class satisfaction, consistent with prior research such as the studies conducted by Baruth and Cohen (2023) and Shih et al. (2014), which demonstrated that personality traits can predict satisfaction in online learning courses among college students. Numerous studies have demonstrated that personality traits are significant factors influencing physical activities (Burgos-Garrido et al., 2011; Wilson et al., 2016; Engels et al., 2022; Piepiora et al., 2023), and they can serve as indicators for predicting sports engagement (Smith et al., 2017) and performance (Piepiora, 2021). The research by Piepiora corroborates the relationship between sports and personality traits: as the duration of physical activities increases, so do the scores on the Big Five personality traits (Piepiora et al., 2022). Personality traits play a role in determining athletic success, interpersonal relationships, and psychological well-being (Piepiora, 2020). Furthermore, distinct sports may exhibit specific associations with particular personality traits, with team sports showing higher scores on Big Five personality indicators (Piepiora, 2021). These findings underscore the pivotal role of personality traits in satisfaction with physical education courses and lend support to the outcomes of this study.

Furthermore, our study indicates a positive correlation between trait fluency and physical education class satisfaction, with trait fluency mediating the relationship between personality traits and physical education class satisfaction. Personality traits significantly predict physical education class satisfaction through the mediation of trait fluency, and trait fluency itself also significantly predicts physical education class satisfaction, confirming its importance as a determinant of class satisfaction (Hu et al., 2008). Consistent with the theory of flow, which posits that the experience of flow is driven by intrinsic motivation (Mandigo and Holt, 2006), individuals engaged in activities driven by intrinsic motivation tend to demonstrate greater engagement and satisfaction (Jackson, 1992). Intrinsic motivation has been found to be positively associated with participation in physical activities (Li, 2010), and extensive research has highlighted trait fluency as an essential factor in physical activities (Yu and Hu, 2009; Gao, 2010; Qu et al., 2017). Moreover, intrinsic motivation is enhanced with an increase in the frequency of trait fluency experiences (Wang et al., 2011). Based on these analyses, our study posits that trait fluency can stimulate intrinsic motivation in physical activities, leading to a greater tendency for highly trait fluent college students to persist in exercising during physical education classes, resulting in higher levels of class satisfaction.

Our research findings confirm the moderating role of physical education class difficulty in the direct and indirect associations between personality traits and physical education class satisfaction. Specifically, we observed a significant negative correlation between physical education class difficulty and class satisfaction, consistent with the findings of Lee and Nuatomue (2021) and Sutherland et al. (2019). The moderated mediation test showed that as the level of physical education class difficulty increased, the predictive effect of personality traits on trait fluency and physical education class satisfaction decreased significantly, while the predictive effect of trait fluency on physical education class satisfaction increased significantly. This indicates that as the level of physical education class difficulty increases, personality traits are relatively more likely to enhance their physical education class satisfaction through trait fluency. The reason behind this could be that certain college students’ trait fluency does not align well with the difficulty level of public physical education classes, making it more likely for them to disengage when faced with higher levels of difficulty. On the other hand, individual students with higher trait fluency may find it easier to experience flow states and match the higher difficulty levels. Similar research seems to support this notion, as demonstrated by Wang et al. (2020), where learning difficulty, as a moderating variable, significantly predicted classroom negativity. Both low and high learning difficulty levels resulted in negative outcomes (Daschmann et al., 2014; Cui et al., 2017), but higher learning difficulty was more likely to induce negative emotions (Tanaka and Murayama, 2014). Therefore, only an appropriate level of difficulty in physical education can promote students to achieve trait fluency and enhance satisfaction in physical education.

This study has certain limitations. Firstly, it adopts a cross-sectional design, which precludes the establishment of causal relationships. For instance, conducting experimental interventions targeting trait fluency and physical education difficulty, altering teaching methods and content, could be employed to ascertain whether improvements in student satisfaction occur. Furthermore, monitoring changes in university students’ physical education satisfaction over time could provide validation of the mediating and moderating effects. Future research should employ longitudinal or experimental designs to confirm the causal hypotheses proposed in this study. Secondly, the limited representativeness of the sample, which is predominantly concentrated in the Shanghai region, hinders the generalizability of the findings. Subsequent research should broaden the sample size to investigate university students from diverse regions and cultural backgrounds, aiming to explore and validate the proposed model. Third, this study exhibits a significant gender imbalance, and future research should consider a more balanced distribution of sexes. Fourth, due to the impact of the COVID-19 pandemic, data collection occurred through a mixed online and offline approach. Despite measures taken to ensure quality and reliability, potential biases cannot be entirely ruled out. Subsequent research should contemplate data collection in more typical circumstances. Fifth, we did not analyze the impact of the five dimensions of personality traits on satisfaction with physical education courses. Subsequent studies delving into the influence of each dimension on satisfaction may offer more insights.

The results of this study can offer guidance to educational policymakers concerning how to enhance university physical education programs to improve student satisfaction. By highlighting the significance of trait flow and the difficulty of physical education courses, school administrators can utilize these findings to enhance their institution’s physical education programs and better cater to student needs. Additionally, these results provide a research framework for other researchers to further investigate practical approaches for personalized improvements in physical education courses.

## 5 Conclusion

This study examines the influence of personality traits on physical education satisfaction among Chinese university students and reveals the mediating role of trait fluency in this relationship. Furthermore, it investigates the moderating effect of physical education difficulty in the pathway between personality traits and physical education satisfaction. For college students, trait fluency enhances physical education satisfaction, and an appropriate level of physical education difficulty serves to further elevate satisfaction by moderating trait fluency. This study provides a theoretical foundation for improving physical education programs and enhancing student satisfaction in these courses.

## Data availability statement

The raw data supporting the conclusions of this article will be made available by the authors, without undue reservation.

## Ethics statement

The studies involving humans were approved by the Ethics Committee of Shandong University. The studies were conducted in accordance with the local legislation and institutional requirements. The participants provided their written informed consent to participate in this study.

## Author contributions

ZC: Conceptualization, Investigation, Methodology, Project administration, Writing – original draft, Writing – review & editing. YT: Investigation, Supervision, Writing – review & editing. ML: Formal analysis, Writing – review & editing. SY: Conceptualization, Funding acquisition, Methodology, Writing – review & editing.
