# Supplementary material for: Personality traits and Chinese college students’ satisfaction with physical education classes: the mediating role of trait fluency and the moderating role of physical education class difficulty
Source: Front Psychol. 2023 Dec 20;14:1270089. doi: 10.3389/fpsyg.2023.1270089 (PMC10762447; doi:10.3389/fpsyg.2023.1270089)
Supplement: Supplementary file 1 [file Data_Sheet_1.docx]

# Supplementary material 1: Physical Education Satisfaction Scale

In this study, we utilized the Youth Physical Exercise Satisfaction Scale, substituting "physical exercise" with "physical education classes," to create a unidimensional structure for the Physical Education Satisfaction Scale comprising five items. Item analysis (Table 1) confirmed the good discriminative ability among the items, allowing for factor analysis.

**Table 1** Item Analysis of the Physical Education Satisfaction Scale (N=62)

| Subject | | Hypothesis | F | Significance | t | Degree of freedom | Sig. (two-tailed) |
| --- | --- | --- | --- | --- | --- | --- | --- |
|  |  |  |  |  |  |  |  |
| Satisfaction 1 | AQE | | 3.616 | 0.066 | -7.372 | 33.000 | 0.000 |
|  | NAQE | |  |  | -7.232 | 22.231 | 0.000 |
| Satisfaction 2 | AQE | | 15.580 | 0.000 | -7.976 | 33.000 | 0.000 |
|  | NAQE | |  |  | -7.823 | 22.049 | 0.000 |
| Satisfaction 3 | AQE | | 11.704 | 0.002 | -6.731 | 33.000 | 0.000 |
|  | NAQE | |  |  | -6.582 | 20.123 | 0.000 |
| Satisfaction 4 | AQE | | 3.616 | 0.066 | -7.372 | 33.000 | 0.000 |
|  | NAQE | |  |  | -7.232 | 22.231 | 0.000 |
| Satisfaction 5 | AQE | | 2.749 | 0.107 | -5.545 | 33.000 | 0.000 |
|  | NAQE | |  |  | -5.473 | 26.572 | 0.000 |
| AQE: assuming equal variance, NAQE: not assuming equal variance | | | | | | | |

According to Table 2, the KMO (Kaiser-Meyer-Olkin) value for the scale is 0.874, indicating its suitability for factor analysis. We employed the principal component analysis method of factor analysis to analyze the Physical Education Satisfaction Scale. One common factor with eigenvalues greater than 1 was extracted, representing physical education satisfaction, with items ranging from satisfaction 1 to satisfaction 5, consistent with the original scale structure. Moreover, the cumulative explained variance of the factor structure accounted for 92.13%, exceeding the threshold of 60%. The factor loadings for each item were all above 0.5, suggesting good structural validity for this scale.

**Table 2** Exploratory Factor Analysis of the Physical Education Satisfaction Scale (N=62)

| Subject | | Factor loadings |
| --- | --- | --- |
| Satisfaction 1 | 0.968 | |
| Satisfaction 2 | 0.964 | |
| Satisfaction 3 | 0.979 | |
| Satisfaction 4 | 0.976 | |
| Satisfaction 5 | 0.910 | |
| Eigenvalues | 4.607 | |
| Cumulative Explained Variance | 92.130 | |
| KMO Sample Suitability Quantity | 0.874 | |
| Extraction method: principal component analysis. | | |

Verified by confirmatory factor analysis (CFA) as shown in Figure 1, the fit indices for the scale are as follows: RMSEA = 0.38 (<0.8), AGFI = 0.984, GFI = 0.995, NFI = 0.996, and RFI = 0.991, all exceeding the threshold of 0.9. The standardized factor loadings of the measurement indicators range from 0.55 to 0.88, meeting the criteria for model adaptability. This indicates a good model fit and further affirms the strong structural validity of the Physical Education Satisfaction Scale.

**Figure 1** The standardized modified model diagram of the confirmatory factor analysis for the Physical Education Satisfaction Scale (N=868)


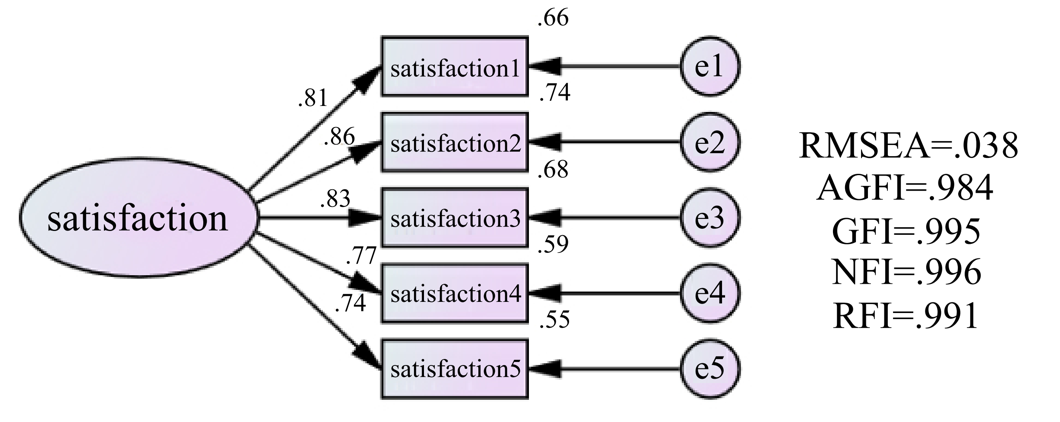


As can be seen from Table 3, the internal consistency reliability Alpha coefficient value of the Big Five Personality Scale is equal to 0.899, and the standardized internal consistency reliability Alpha coefficient value is equal to 0.900, which is a desirable indicator of reliability, indicating that the internal consistency of the scale is high, i.e., the reliability of the scale is good.

**Table3** Reliability Analysis of the Physical Education Satisfaction Scale (N=868)

| Meters | Alpha | Standardized Alpha | Item count |
| --- | --- | --- | --- |
| PE Satisfaction | 0.899 | 0.900 | 5 |

# Supplementary material 2: Physical Education Difficulty Scale

In this study, we employed the MOOC Difficulty Scale, with the term "MOOC" replaced by "Physical Education Class," to create a unidimensional scale known as the Physical Education Difficulty Scale, consisting of three items. Item analysis (Table 4) demonstrates adequate discriminant validity among the scale items, thus allowing for factor analysis.

**Table 4** A glance at the item analysis of the Physical Education Difficulty Scale (N=62)

| Subject | Hypothesis | F | Significance | t | Degrees of freedom | Sig. (two-tailed) |
| --- | --- | --- | --- | --- | --- | --- |
| Difficulty 1 | AQE | 0.941 | 0.339 | 2.622 | 33.000 | 0.013 |
|  | NAQE |  |  | 2.640 | 31.979 | 0.013 |
| Difficulty 2 | AQE | 3.095 | 0.088 | 2.131 | 33.000 | 0.041 |
|  | NAQE |  |  | 2.151 | 30.792 | 0.039 |
| Difficulty 3 | AQE | 2.030 | 0.164 | 2.289 | 33.000 | 0.029 |
|  | NAQE |  |  | 2.312 | 30.376 | 0.028 |

From Table 5, it can be observed that the KMO value of this scale is 0.755. We conducted a factor analysis using the principal component analysis method on the Physical Education Class Difficulty Scale. One common factor was extracted with eigenvalues greater than 1, representing the construct of physical education class difficulty. The items corresponding to this factor are labeled as Difficulty 1 to Difficulty 3 (Q10_1 to Q10_3), aligning with the original scale structure. Additionally, the cumulative explained variance by the factor construct is 88.517%, exceeding the 60% threshold, and the factor loadings for each item are all above 0.5. These preliminary findings suggest that the scale possesses good structural validity.

**Table 5** Exploratory Factor Analysis of the Physical Education Difficulty Scale (N=62)

| Subject | Factor loadings |
| --- | --- |
| Difficulty 1 | 0.956 |
| Difficulty 2 | 0.931 |
| Difficulty 3 | 0.935 |
| Eigenvalues | 2.655 |
| Cumulative Explained Variance | 88.517 |
| KMO Sample Suitability Quantity | 0.755 |
| Extraction method: principal component analysis. | |

In the confirmatory factor analysis (CFA), as shown in Figure 2, the fit indices for the scale are presented in the diagram. The results indicate that this model is a saturated model. According to the definition of a saturated model, it has zero degrees of freedom, meaning it imposes the least artificial constraints and is constructed based on the interrelationships within the data, thus representing the optimal model. Mathematically, it is an ideal state model that can perfectly fit observed data. This further underscores the excellent structural validity of the Physical Education Class Satisfaction Scale.


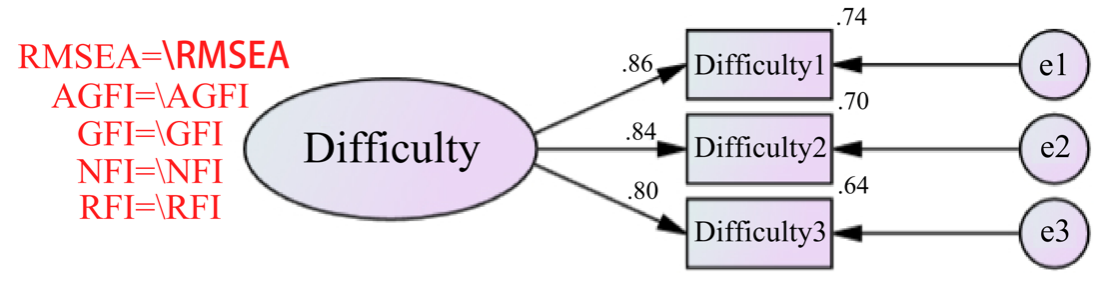
**Figure 2** The standardized modified model diagram of the confirmatory factor analysis for the Physical Education Difficulty Scale (N=868)

From Table 6, it can be observed that the internal consistency reliability of the Flow Trait Scale is indicated by an Alpha coefficient value of 0.870, while the standardized internal consistency reliability Alpha coefficient value is 0.871. These reliability indicators are excellent, demonstrating a high level of internal consistency for the scale. In other words, the scale exhibits strong reliability.

**Table 6** Reliability analysis of the Trait Fluency Scale at a glance (N=868)

| Meters | Alpha | Standardized Alpha | Item count |
| --- | --- | --- | --- |
| PE Difficulty | 0.870 | 0.871 | 3 |
